# Isolation and identification of antimicrobial compound from *Mentha longifolia *L. leaves grown wild in Iraq

**DOI:** 10.1186/1476-0711-8-20

**Published:** 2009-06-12

**Authors:** Firas A Al-Bayati

**Affiliations:** 1 Department of Biology, College of Education, University of Mosul, Mosul, Iraq

## Abstract

**Background:**

*Mentha longifolia *L. (Lamiaceae) leaves have been traditionally implemented in the treatment of minor sore throat and minor mouth or throat irritation by the indigenous people of Iraq, although the compounds responsible for the medicinal properties have not been identified. In the present study, an antimicrobial compound was isolated and characterized, and its biological activity was assessed.

**Methods:**

The compound was isolated and characterized from the extracted essential oil using different spectral techniques: TLC, FTIR spectra and HPLC. Antimicrobial activity of the compound was assessed using both disc diffusion and microdilution method in 96 multi-well microtiter plates.

**Results:**

A known compound was isolated from the essential oil of the plant and was identified as (-) menthol. The isolated compound was investigated for its antimicrobial activity against seven selected pathogenic and non-pathogenic microorganisms: *Staphylococcus aureus, Streptococcus mutans, Streptococcus faecalis, Streptococcus pyogenis, Lactobacillus acidophilus, Pseudomonas aeruginosa *and the yeast *Candida albicans*. Menthol at different concentrations (1:1, 1:5, 1:10, 1:20) was active against all tested bacteria except for *P. aeruginosa*, and the highest inhibitory effect was observed against *S. mutans *(zone of inhibition: 25.3 mm) using the disc diffusion method. Minimal inhibitory concentration MIC values ranged from 15.6–125.0 μg/ml, and the most promising results were observed against *S. aureus *and *S. mutans *(MIC 15.6 μg/ml) while, *S. faecalis, S. pyogenis *and *L. acidophilus *ranked next (MIC 31.2 μg/ml). Furthermore, menthol achieved considerable antifungal activity against the yeast *C. albicans *(zone of inhibition range: 7.1–18.5 mm; MIC: 125.0).

**Conclusion:**

The isolation of an antimicrobial compound from *M. longifolia *leaves validates the use of this plant in the treatment of minor sore throat and minor mouth or throat irritation.

## Background

Many infectious diseases are known to be treated with herbal remedies throughout the history of mankind. Even today, plant materials continue to play a major role in primary health care as therapeutic remedies in many developing countries. Plants still continue to be almost the exclusive source of drugs for the majority of the world's population. The World Health Organization reported that 80% of the worlds population rely chiefly on traditional medicine and a major part of the traditional therapies involve the use of plant extracts or their active constituents [1].

*Mentha longifolia *L. (common name: wild mint or horse mint) member of the large mint family Lamiaceae, is a fast-growing, perennial herb which can reach up to 1.5 m high in favourable conditions. *M. longifolia *is an extremely variable species with a widespread distribution in Iraq, Mediterranean region, Europe and eastwards into Asia. In Iraqi folk medicine, the leaves are used for relief of minor sore throat and minor mouth or throat irritation. It is also used in treatments for minor aches and sprains, and in nasal decongestants. In addition to its antipruritic, carminative, antiseptic and stimulant properties [2].

Menthol (C_10_H_20_O) is a terpenoid, found in the essential oils of the mint family (*Mentha *spp.), such as peppermint, horse mint and others. It is a waxy, crystalline substance, clear or white in colour, which is solid at room temperature and melts slightly above. Several isomers of menthol exist, some with a menthol smell, others without. In nature it only occurs as (-) menthol, which has the strongest smell and its formal name is (1R,2S,5R)-2-isopropyl-5-methylcyclohexanol. The other isomers are known as isomenthol, neomenthol and neoisomenthol.

(-) menthol can be described as fresh, sweet, minty, cooling, refreshing. The (+) isomer is similar, but less minty, more herby, with musty, bitter, phenolic and herbaceous notes, and is less refreshing. (-) menthol has also got about four times the cooling power of the (+) isomer [3].

In spite of all the information available in literature, no extensive isolation studies of (-) menthol are present. Thus, the aim of this study was to isolate and characterize menthol from *M. longifolia *grown in Iraq using different spectral techniques, and its antimicrobial activity against some selected pathogenic and non-pathogenic microorganisms.

## Methods

### Chemicals and Reagents

Potassium hydroxide KOH, diethyl ether (CH_3_CH_2_)_2_O, carbon disulfide CS_2_, heptane CH_3_(CH_2_)_5_CH_3_, methanol CH_3_OH and dimethylsulfoxide "DMSO" were supplied from BDH Analar (England). (-) Menthol (standard) ≥ 99% purity (molecular weight 156.27, mp 41–44°C, bp 212°C, molecular formula C_10_H_20_O) and *p*-iodonitrotetrazolium violet (INT) were obtained from Sigma-Aldrich Chemical Company.

### Plant material

*Mentha longifolia *leaves were obtained commercially from a local market in Mosul city, Nineveh province, Iraq and identified by a botanical taxonomist at college of Agriculture and Forestry, University of Mosul. The leaves were washed first under running tap water, followed by sterilized distilled water and dried at room temperature in dark then grinded to powder using an electrical blender

### Essential oil extraction and isolation of (-) menthol

The dried plant material was submitted to steam distillation in a Clevenger-type apparatus for 3 h. A volume of 1.0 ml (density = 1.04 g/ml) of the resulted plant essential oil was dissolved with 50 ml of heptane and transferred to a 125 ml separatory funnel. 25 ml of methanol was added. The funnel was shaken vigorously and the layers were separated. The heptane phase was dried over anhydrous sodium sulphate to produce menthol [4].

### Characterization of (-) menthol

#### Chemical detection

Chemical detection was carried out by adding a small piece of potassium hydroxide into a test tube containing 1 ml of the plant essential oil with heating. The solution was cooled and 1 ml of diethyl ether was added. A few drops of carbon disulfide was added to the solution forming a yellow residue that indicates the presence of menthol [4].

#### Thin-layer chromatography (TLC)

The isolated compound was dissolved in appropriate solvent. 5 μl of reference solution of menthol and 5 μl of investigated wild mint oil were applied to silica gel plates, Merck (Germany) 20 × 20 cm, 0.25 mm in thickness. Plates were developed using the solvent system toluene: diethyl ether: 1.75 M acetic acid (1:1:1) and the separated zones were visualized using iodine I2. Standard menthol served as positive control.

#### FTIR studies

The IR spectrum of menthol was recorded in the College of Education, Department of Chemistry, University of Mosul, using a computerized Tensor 27 FTIR spectrometer (Bruker Co., Germany) in the range of 400–4000 cm-1 by the KBr pellet technique.

#### High-performance liquid chromatography (HPLC)

HPLC analysis was performed in College of Science, University of Mosul, using a Shimadzo LC 2010 HPLC system (Kyoto, Japan), equipped with a Shimadzo LC 2010 UV-VIS detector with a thermostatted flow cell and a selectable two wavelengths of 190–370 nm or 371–600 nm. The detector signal was recorded on a Shimadzo LC 2010 integrator. The column used was a C18 block heating-type Shim-pack VP-ODS (4.6 mm interior diameter × 150 mm long) with a particle size of 5 μm. Menthol was separated using a mobile phase of 3% ethyl acetate/isooctane at a flow rate of 3.0 ml/min, column temperature 25°C. Injection volume was 40 μl and detection was carried out at 322 nm.

### Antimicrobial activity

#### Microbial cultures

Six strains of bacteria and one yeast were used as test microorganisms. The bacterial strains included Gram-positive *Staphylococcus aureus*, *Streptococcus mutans*, *Streptococcus faecalis*, *Streptococcus pyogenis *and *Lactobacillus acidophilus*: Gram-negative *Pseudomonas aeruginosa*; and the yeast *Candida albicans*. All microorganisms were clinical isolates, obtained from the Microbiology Laboratory at Department of Basic Science, College of Nursing, University of Mosul, Iraq.

#### Inoculum preparation

Nutrient broth and Sabouraud dextrose agar (SDA) were used for growing and diluting the microorganism suspensions. Bacterial strains were grown to exponential phase in nutrient broth at 37°C for 18 h and adjusted to a final density of 10^8 ^cfu/ml by diluting fresh cultures and comparison to McFarland density. *C. albicans *was aseptically inoculated on petri dishes containing autoclaved, cooled, and settled SDA medium. The petri dishes were incubated at 31°C for 48 h to give white round colonies against a yellowish background. These were aseptically subcultured on SDA slants. The yeast colonies from SDA slants were suspended in sterilized 0.9% sodium chloride solution (normal saline), which was compared with McFarland solution. According to the manufacturer's directions, 1 ml of yeast suspension in normal saline was added to 74 ml of sterile medium and kept at 45°C to give a concentration of 2 × 10^7 ^cells/ml.

#### Disc diffusion assay

A modified agar diffusion method [5] was used to determine antimicrobial activity. Nutrient agar was inoculated with microbial cell suspension (200 μl in 20 ml medium) and poured into sterile petri dishes. Sterile filter paper discs 6 mm in diameter were impregnated with 20 μl of menthol in different concentrations (1:1, 1:5, 1:10, 1:20 initially prepared by diluting in DMSO and sterilized by filtration through 0.45 μm millipore filters), and placed on the inoculated agar surface. Standard 6 mm discs containing streptomycin 25 μg/disc and amphotericin B 10 μg/disc (Bioanalyse) were used as positive controls. The plates were incubated overnight at 37°C for 18–24 h. In contrast, *C. albicans *was incubated at 31°C for 48 h. and the diameter of any resulting zones of growth inhibition was measured (mm). Each experiment was tested in triplicate.

#### Micro-well dilution assay

Minimal inhibitory concentrations of menthol isolated from *M. longifolia *was determined based on a microdilution method in 96 multi-well microtiter plates as previously described [6]. Briefly, bacterial strains were cultured overnight at 37°C on nutrient broth and adjusted to a final density of 10^8 ^cfu/ml, and used as an inoculum. Menthol was dissolved in DMSO and then in nutrient broth to reach a final concentration of 500.0 μg/ml. Serial twofold dilutions were made in a concentration range from 7.8 to 500.0 μg/ml. In each microtiter plate, a column with broad-spectrum antibiotic was used as positive control (streptomycin in serial dilutions 500.0-7.8 μg/ml). As an indicator of bacterial growth, 50 μl of 0.2 mg/ml *p*-iodonitrotetrazolium chloride (INT) was added to the wells and incubated at 37°C for 30 min. The lowest concentration of compound showing no growth was taken as its minimal inhibitory concentration MIC. The colourless tetrazolium salt acts as an electron acceptor and is reduced to a red-coloured formazan product by biologically active organisms [7]. Where bacterial growth was inhibited, the solution in the well remained clear after incubation with INT.

As for *C. albicans*, a simple turbidity test [8] was used to determine the MIC value of menthol. A volume of 0.1 ml from each serial dilution of menthol concentrations (500.0-7.8 μg/ml) was added into tubes containing 9.8 ml of sterile nutrient broth, and then the tubes were inoculated with 0.1 ml of yeast suspension and incubated at 31°C for 48 h. Amphotericin B (500.0-7.8 μg/ml) was used as a positive control. The optical density was determined using a Spectro SC spectrophotometer (LaboMed, Inc.) at 630 nm. The MIC value was the lowest concentration of compound that showed no growth after 48 h of incubation in comparison with the control tube, which included 9.8 ml of nutrient broth and 0.1 ml of yeast suspension in addition to 0.1 ml of each compound concentration (unincubated).

## Results

**Figure 1:**
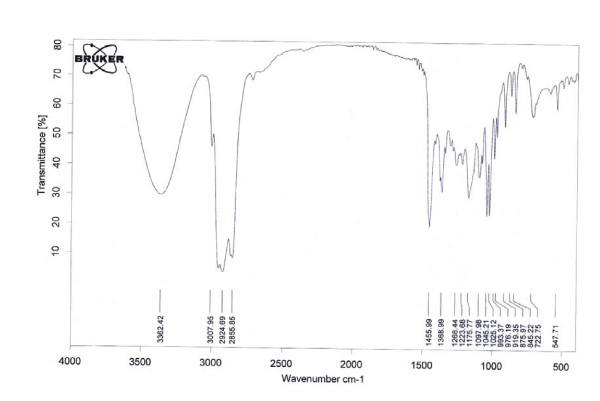
**FTIR spectrum of menthol isolated from *M. longifolia *leaf oil**.

**Figure 2:**
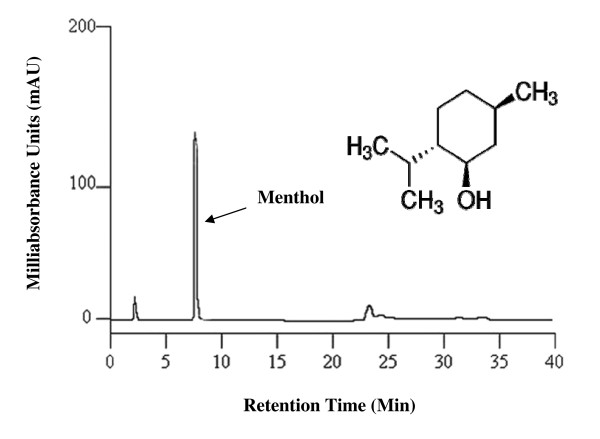
**HPLC chromatogram of menthol isolated from *M. longifolia *leaf oil**.

The present study was conducted to isolate the main bioactive compound from *M. longifolia *leaf oil. Menthol was isolated from the extracted essential oil, and then detected on TLC plates in comparison with standard menthol. A purple zone with a retention factor (*R*_*f*_) value of 0.62 was identified as menthol in comparison with standard menthol that had the same *R*_*f *_value. The FTIR spectrum confirmed the material isolated from *M. longifolia *leaf oil as menthol (Fig. 1). Significant peaks were found at: (3362) cm^-1 ^corresponding to hydroxyl group; (2855, 2924) cm^-1 ^ascribed to methyl group; (1025, 1045) cm^-1 ^attributed to (C-O) bond and (1368) cm^-1 ^corresponding to isopropyl group, all of which confirm the purity of the isolated material. Moreover, menthol was characterized using the HPLC system (Fig. 2) and identified by comparing its retention time (*t*_*R*_) and UV spectrum with that of the standard compound. The retention time 7–8 min and UV spectra of the isolated compound on HPLC were completely identical to that of standard menthol.

The isolated compound was investigated for its antimicrobial activity against six bacterial species and one yeast. The initial screening of antibacterial activity of menthol was assayed *in vitro *by the agar diffusion method using four concentrations (1:1, 1:5, 1:10, 1:20). All menthol concentrations were active against all tested bacteria except for *P. aeruginosa *(Table 1). The highest inhibitory effect was observed against *S. mutans *(zone of inhibition: 25.3 mm) using the concentration (1:1), while the weakest activity was demonstrated against *S. pyogenis *(zone of inhibition: 10.0 mm) using the concentration (1:20). In view of the results obtained by the disc diffusion method, the minimal inhibitory concentration MIC of menthol was determined by broth microdilution assay (Table 2). The highest MIC value (15.6 μg/ml)was observed against *S. aureus *and *S. mutans*, while *S. faecalis*, *S. pyogenis *and *L. acidophilus *ranked next (MIC 31.2 μg/ml). Moreover, menthol observed good antifungal activity against the yeast *C. albicans *(zone of inhibition range: 7.1–18.5 mm; MIC: 125.0).

**Table 1:** Antimicrobial activity of menthol isolated from *M. longifolia *leaf oil.

Microorganisms	Zone of inhibition (mm)					
	
	Menthol concentrations				Control	
	
	1:1	1:5	1:10	1:20	S	A
*S. aureus*	24.8	20.6	15.7	11.8	20.9	N.T
*S. mutans*	25.3	21.9	17.5	12.3	19.5	N.T
*S. faecalis*	23.2	19.2	15.0	10.7	24.3	N.T
*S. pyogenis*	22.3	18.3	14.8	10.0	19.2	N.T
*L. acidophilus*	23.6	19.3	15.4	10.5	18.1	N.T
*P. aeruginosa*	00.0	00.0	00.0	00.0	11.2	N.T
*C. albicans*	18.5	15.4	11.0	7.1	N.T	10.2

S: Streptomycin (25 μg/disc), A: Amphotericin B (10 μg/disc), N.T: Not tested

**Table 2:** Minimum inhibitory concentration (MIC) of menthol isolated from *M. longifolia *leaf oil.

Microorganisms	MIC values (μg/ml)		
	
	Menthol	Control	
		
		S	A
*S. aureus*	15.6	7.8	N.T
*S. mutans*	15.6	7.8	N.T
*S. faecalis*	31.2	7.8	N.T
*S. pyogenis*	31.2	7.8	N.T
*L. acidophilus*	31.2	7.8	N.T
*P. aeruginosa*	>500.0	15.6	N.T
*C. albicans*	125.0	N.T	7.8

S: Streptomycin, A: Amphotericin B, N.T: Not tested

The standard drug streptomycin was active against all reference bacteria (zone of inhibition range: 11.2–24.3 mm; MIC range: 15.6–7.8 μg/ml). In addition, amphotericin B demonstrated good antifungal activity against *C. albicans *(zone of inhibition: 10.2 mm; MIC: 7.8 μg/ml).

## Discussion

The fragrance of plants is carried in the so called quinta essentia, or essential oil (EO) fraction. These oils are secondary metabolites that are highly enriched in compounds based on an isoprene structure. They are called terpenes, their general chemical structure is C_10_H_16_, and they occur as diterpenes, triterpenes, and tetraterpenes (C_20_, C_30_, and C_40_), as well as hemiterpenes (C_5_) and sesquiterpenes (C_15_). When the compounds contain additional elements, usually oxygen, they are termed terpenoids such as menthol and camphor [9].

*In vitro *studies in this work showed that menthol inhibited the growth of all tested bacteria except *P. aeruginosa *and observed good antifungal activity against the yeast *C. albicans*. The zones of inhibition ranged from 10.0–25.3 mm and 7.1–18.5 mm in diameter against *C. albicans *using the disc diffusion method. Furthermore, MIC values ranged from 15.6–31.2 μg/ml against tested bacteria and 125.0 μg/ml against *C. albicans*. Terpenenes or terpenoids have been previously reported to be active against bacteria [10,11], fungi [12,13], viruses [14,15], and protozoa [16,17]. The mechanism of action of terpenes is not fully understood but is speculated to involve membrane disruption by the lipophilic compounds [9].

In view of the results obtained using both disc diffusion and micro-well dilution assays, menthol was found only active against Gram-positive bacteria. It has frequently been reported that Gram-positive bacteria are more susceptible to essential oils than Gram-negative bacteria [18]. The tolerance of Gram-negative bacteria to essential oils has been ascribed to the presence of a hydrophilic outer membrane that blocks the penetration of hydrophobic essential oils into target cell membrane.

The Gram-negative bacterium *P. aeruginosa *resisted all menthol concentrations and was only inhibited using the standard drug. Several studies have reported that the Gram-negative bacteria, *Pseudomonas*, and in particular *P. aeruginosa*, appear to be least sensitive to the action of essential oils [19,20]. Furthermore, Several mechanisms of antimicrobial resistance are readily spread to a variety of bacterial genera. First, the organism may acquire genes encoding enzymes, such as β-lactamases, that destroy the antibacterial agent before it can have an effect. In addition, bacteria may acquire efflux pumps that extrude the antibacterial agent from the cell before it can reach its target site and exert its effect. Finally, bacteria may acquire several genes for a metabolic pathway which ultimately produces altered bacterial cell walls that no longer contain the binding site of the antimicrobial agent, or bacteria may acquire mutations that limit access of antimicrobial agents to the intracellular target site via down-regulation of porin genes [21].

## Conclusion

A number of EO components have been registered as flavourings in foodstuffs. The flavourings registered are considered to present no risk to the health of the consumer and include amongst others carvacrol, carvone, cinnamaldehyde, citral, p-cymene, eugenol, limonene, menthol and thymol [22]. In the present study, the isolated compound demonstrated promising antimicrobial activities against the most prevalent microorganisms in oral infections. The use of this plant in the treatment of sore throat, mouth or throat irritation is validated, scientifically supported by the results obtained in this work.

## Competing interests

The author declares that they have no competing interests.
